# Is It Feasible to Apply a Virtual Box and Block Test in Children with Unilateral Cerebral Palsy?: A Pilot Study

**DOI:** 10.3390/jcm14020391

**Published:** 2025-01-09

**Authors:** Soraya Pérez-Nombela, Javier Merino-Andrés, Julio Gómez-Soriano, María Álvarez-Rodríguez, Silvia Ceruelo-Abajo, Purificación López-Muñoz, Rocío Palomo-Carrión, Ana de los Reyes-Guzmán

**Affiliations:** 1 Toledo Physiotherapy Research Group (GIFTO), Faculty of Physiotherapy and Nursing, Universidad de Castilla-La Mancha, 45071 Toledo, Spain; soraya.perez@uclm.es (S.P.-N.); julio.soriano@uclm.es (J.G.-S.); 2 Toledo Physiotherapy Research Group (GIFTO), Instituto de Investigación Sanitaria de Castilla-La Mancha (IDISCAM), 45004 Toledo, Spain; 3 Biomechanics and Technical Aids Unit, Hospital Nacional de Parapléjicos, 45004 Toledo, Spain; maria.alvarez.rodriguez93@gmail.com (M.Á.-R.); adlos@sescam.jccm.es (A.d.l.R.-G.); 4 Rehabilitation Department, Hospital Nacional de Parapléjicos, 45004 Toledo, Spain; sceruelo@sescam.jccm.es; 5 Improve-Lab, Faculty of Physiotherapy and Nursing, Universidad de Castilla-La Mancha, 45071 Toledo, Spain; purificacion.lopez@uclm.es (P.L.-M.); rocio.palomo@uclm.es (R.P.-C.); 6 Unidad de Neurorrehabilitación, Biomecánica y Función Sensitivo-Motora (HNP-SESCAM), Unidad Asociada de I+D+I al CSIC, 45004 Toledo, Spain

**Keywords:** children, rehabilitation, Leap Motion Controller, virtual reality, manual dexterity

## Abstract

**Background**: With technological advancements, virtual versions of the Box and Block Test (BBT) employing the Leap Motion Controller have been developed for evaluating hand dexterity. Currently, there are no studies about the usefulness of this system in children with unilateral cerebral palsy (UCP). Thus, our main objective is to apply a virtual BBT based on the Leap Motion Controller in children with UCP compared with the real BTT for assessing upper limb function within a pilot study. **Methods:** Seven children between the ages of 4 and 8 years who were diagnosed with UCP were assessed three times using the real and virtual BBT. **Results:** For all the participants, performance was greater in the real BBT than in the virtual BBT. During the last assessment, the participants reached 28.17 (SD:6.31) blocks in the real test and 9.00 (SD:5.90) in the virtual test. The correlation index between the two modalities of the BBT was moderate (r = 0.708). **Conclusions:** The results obtained in this study suggest that the application of the virtual BBT in children with UCP is feasible. Future studies are needed to validate the application of the virtual BBT in children with UCP.

## 1. Introduction

Cerebral palsy (CP) is defined as a group of permanent developmental disorders that affect movement and posture, with implications for body structure and function, as well as for activity and participation [1]. It is caused by non-progressive alterations in the development of the fetal or infant brain [2]. CP is one of the leading causes of motor disability in children, with a birth prevalence in high-income countries of 1.5 per 1000 live births [3].

Unilateral cerebral palsy (UCP) is the most common subtype of CP and accounts for 20% to 40% of new cases [4]. Children with UCP experience upper limb dysfunctions, the severity of which can vary depending on the timing, location, extent and nature of the brain injury. However, the most relevant aspects are spasticity, weakness and loss of motor coordination in the affected upper limb [5], which can translate into impaired upper limb function (unimanual and bimanual), affecting independence, participation and quality of life [6] in each child’s natural environment [7].

In this context, upper limb rehabilitation in children with UCP plays a fundamental role in improving independence, participation and quality of life. A meta-analysis of the effectiveness of therapies for functional recovery of the upper limb revealed moderate evidence that intensive, task-oriented interventions, such as bimanual training, are more effective than traditional therapies [6].

On the other hand, effective evaluation of children with chronic health conditions, such as UCP, has a very important role in monitoring their progress or evaluating any type of intervention [8]. Thus, the assessment of manual dexterity is an important indicator of upper limb function, and both clinicians and researchers frequently measure it to assess the effectiveness of the rehabilitation process.

The Box and Block Test (BBT) was developed by Mathiowetz and is designed to measure manual dexterity in an easy, quick and simple way without the need for a specific setting [9]. Recently, the properties of this scale have been measured for children with UCP, demonstrating that the BBT is a reliable and valid tool for this population and that, both for research and clinical applications, an improvement of six blocks is recommended in this test as the minimal clinically important difference (MCID) [9].

Owing to the growing interest in and use of technology in the field of neurorehabilitation, virtual reality (VR) has become a relevant complement in the rehabilitation of motor skills and functional development in children with UCP. With respect to the improvements that VR can bring to the manual function of children with UCP, improvements can be found in finger dissociation, grasp, weight bearing [10] and the quality of functional movement [11], thereby increasing manual dexterity. However, further research is needed to evaluate the various components of manual function within the application of VR. Virtual environments can replace assessment or treatment in situations where real-world application is difficult, in addition to offering a highly motivating environment for children where they can perform activities or tasks comparable to real situations. Moreover, the application of virtual BBT could address the issues related to shapes and surfaces presented by real BBT [12,13], thereby allowing the evaluation of other aspects of manual function that could be affected by sensory alterations due to UCP [14], which might otherwise distort the results of real BBT. Thus, VR appears to be effective both as a rehabilitation tool and for functional assessment [10,15].

It may seem that one of the challenges of using virtual reality systems is the cost of the technology. However, some devices have significantly reduced their prices and are very affordable. Unlike real tests, a virtual application of the BBT can be used for different therapeutic purposes, as it is a powerful tool for training or assessing various functional deficits in the clinical setting [15]. This possibility of using virtual applications with different objectives, along with the ability to obtain objective data from technological devices and increased motivation in patients, is the main reason for developing systems with VR within the field of rehabilitation.

In addition, VR presents another benefit, which is motivation when carrying out certain interventions [16], since it is a fun proposal, which increases adherence to treatment [17], improving functional abilities and skills [18] through the performance of many more repetitions of a certain movement when compared to conventional practice [19].

In this context, the BBT has been developed in a virtual format with low-cost technology, aiming to apply VR as an evaluation of upper limb function both in people without pathology [20] and in people with Parkinson’s disease [15], stroke [21] or spinal cord injury [22]. However, there is no virtual application in the pediatric population, which would include children with UCP.

The main objective of this study was to analyze the feasibility of applying a virtual BBT based on the Leap Motion Controller (LMC) in children with UCP and to analyze the correlation between the virtual BBT and the real clinical scale for assessing upper limb function within a pilot study. Therefore, this study focuses on the process of developing and implementing virtual BBT to address the question of whether this tool could be useful in children with UCP. A secondary objective was to verify whether the scores of the BBT scale, both for the real and virtual versions, may be related to clinical variables in children with UCP.

## 2. Materials and Methods

### 2.1. Participants

The participants in the study were children diagnosed with UCP, who were between the ages of 4 and 8 years (or who would be 4 years old in the year the study started), with levels between I and III on the Manual Ability Classification System (MACS) [23] and with levels between I and III on the Gross Motor Function Classification System (GMFCS) [24]. Additionally, the children were required to have preserved cognitive ability to understand the execution of the proposed activities. The clinical data of the participants are shown in Table 1. All parents or legal representatives of the included children signed informed consent before participating in the study, which was approved by the Ethics Committee for Clinical Research of the Complejo Hospitalario de Toledo (approval number: 372; date: 30 April 2019).

### 2.2. Materials

#### 2.2.1. Box and Block Test

**Figure 1:**
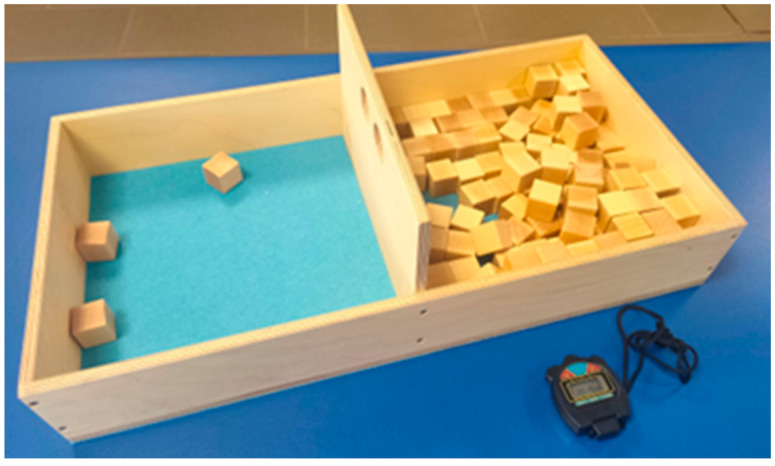
Box and Block test. Real scale with wooden box and cubes.

The Box and Block test was administered to the participants following the specifications previously described by Mathiowetz and colleagues [25]. The scale consists of a wooden box divided into two compartments; a high partition is located between these compartments and 100 wooden cubes of 2.5 cm (Figure 1). The test involves moving the greatest number of blocks from one compartment to another, one at a time, for one minute. The score is the number of blocks that have passed.

#### 2.2.2. Virtual Box and Block Test Based on Leap Motion Controller

The LMC (Leap Motion Controller (Ultraleap, Mountain View, CA, USA) with software (Leap_Motion_Orion_SDK_3.1.0) is a low-cost device that has been designed to control applications via hand gestures and movements. It contains three infrared lights and two cameras. The device is rectangular, 7.3 cm × 3 cm × 1.3 cm (length, width and height), and its weight is 45 g. The LMC is connected to the computer via a USB connection.

For its performance by means of the LMC, a virtual version of the BBT was developed and adapted to neurological patients’ conditions by using the Unity3D game engine and C# programming language. In this study, the characteristics of the computer used were an i7 processor running under Windows 10, with 12 GB of RAM memory and an NVIDIA graphics card.

The virtual BBT is composed of several scenes. The Game scene is the main scene of the virtual application and is composed of the following:

- SceneElements: this includes the scene’s main camera, lights and particle systems.
- LeapElements: elements that allow the capture of hand movements by the Leap Motion sensor.
- Graphical User Interface (GUI).
- WoodBox: formed by the box where the cubes and triggers used in the virtual application will be placed.

The PlayManager script is responsible for controlling the game in general, triggering the events necessary to run the game, and managing changes such as game completion. This includes updating the new data of the level to be played or the graphical interface.

The main component is the model of the box (WoodBox), made via Blender, following the measurements described above in the description of the real scale, and adding a texture imitating wood to maintain the semantics of the original scale. The model is exported in FBX format. One of the peculiarities of this object is that it has kinematic features, i.e., it does not use a physics engine. The box also has an EmptyObject in its hierarchy, called “SourceCubes”, strategically positioned at the point where the cubes will be created.

For the cubes, a prefab has been built in Unity, i.e., a template from which new instances of the object can be created.

This cube prefab is made up of different components, the most important of which are the following: transform, with the scale indicated in the article or by the corresponding level; the physics components boxcollider and rigidbody, the first one creating an object (in this case with the shape of a cube) to detect the collisions that the object has, and the second one adding physics to the object, such as mass or being affected by gravity; and the associated scripts, GrabbableObject and CubeScript, the former provided by the Leap Motion library, which assigns certain properties to the GameObject to allow a better grip, and the latter adding a random material within an array of colors, with a random position and rotation, so that the set of cubes gives a sense of diversity and randomness. It also changes the color of the cube when it is grabbed, which makes it easier to know when it can move.

This script also manages what happens when a cube is on one side or the other of the box and if it overcomes the barrier that separates the two sides, as well as the sound effects of the cubes when they hit the wood or when they score a point.

Another main element is the user interface. It is composed of many elements:

- Instructions panel, with the explanatory videos that are played before the game.
- Elements that compose the countdown of the start of the game (text and progress bar).
- Button and panel for selecting the hand to be used.
- Participant feedback elements: progress bar within the VR as well as the countdown clock.
- Score indicator (number of cubes).
- Summary panel after finishing a level, showing information about the level.
- Save game panel.

The MenuManager object, which is responsible for the management of all the elements mentioned above, has been created.

Continuing with the box, there are three invisible objects, one located on each side of the box and another in the partition of the box and increasing in height. The purpose of these objects is as follows: having no physical properties and taking up no space, all three function as triggers when an object comes into contact with them; an event thus takes place, in this case the counting of points when crossing a cube from one side to the other or adding more cubes if the compartment becomes empty.

LMC elements allow hand models to be represented in the scene. The Unity scene is represented by a model of the sensor and the area it covers. In addition, the virtual model of the hands is shown. PinchingRigidHand and PluginLeapNotice are two elements that allow hand gestures to be as correct as possible.

**Figure 2:**
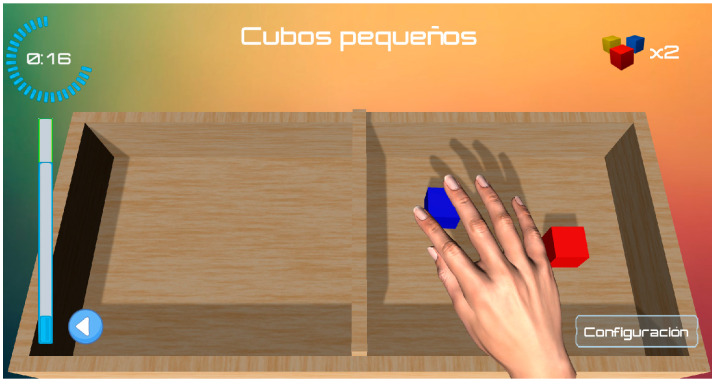
Virtual Box and Block test.

With the aim of facilitating manipulation of the blocks in the virtual environment, only three blocks appear in the box partition at the same time (Figure 2). As the blocks are passed to the other box side, another three blocks appear randomly. The virtual BBT has been designed with multiple difficulty levels on the basis of the cube sizes. To resemble the real test, all the participants performed the virtual test at level 3, where the size of the cubes was similar to that of the real test.

### 2.3. Experimental Setup

The seven participants were measured at three time points (the first point: T1, the second point: T2 and the third point: T3), which were separated by at least three weeks. At each time point, muscle strength was assessed using the International Scale (0–5) for both upper limbs, and hypertonia was assessed using the Ashworth scale for more affected upper limb. Each participant first performed the real BBT and, after a five-minute rest, then performed the virtual BBT. Each participant previously provided proof of habituation to the virtual environment. All evaluations were consistently performed by the same specialists.

Each participant performed both tests seated in a chair in front of the box, with a height-adjustable table so that the participants started with their hands flat on the table. For the BBT, both real and virtual, first, a fifteen-second training trial is allowed for the participant to become familiar with the test, followed by the dominant (or less affected) hand and then the non-dominant (or more affected) hand, See Video S1: Example of performing the real and virtual BBT test.

### 2.4. Data Analysis

To evaluate the correlation between the number of blocks transferred in the real BBT and in the virtual BBT, Spearman’s correlation coefficient was used. Correlations between 0 and 0.25 were considered low; those between 0.25 and 0.5 were considered fair; those between 0.5 and 0.75 were considered moderate; and those greater than 0.75 were considered strong [15,22]. Spearman’s correlation coefficient was also used to evaluate the scores obtained in the real and virtual BBTs with the clinical variables of muscle strength and hypertonia.

Differences in performance between the real and virtual BBTs were analyzed via the Mann–Whitney rank sum test, whereas intragroup differences in strength, hypertonia and BBT (real and virtual) were assessed via the Friedman repeated-measures analysis of variance on ranks and the post hoc Tukey test.

Intraclass correlation coefficients (ICCs) were calculated to evaluate the intrarater reliability of the total score of each evaluation method at different time points. ICC calculation is a proper inferential statistical method for assessing the consistency or absolute agreement among multiple observations made on randomly selected objects of measurement and when the error variance for measures is uniform across the conditions of measurement [26]. According to Shrout et al., the following values are established: an ICC ≤ 0.4 indicates that agreement is weak; an ICC between 0.4 and 0.6, moderate; an ICC between 0.6 and 0.8, good; and an ICC > 0.8, excellent [27]. The confidence interval was 95%, considering that those values of *p* < 0.05 were significant.

Data analysis was carried out with SPSS software for Windows, version 17.0. (SPSS, Chicago, IL, USA).

## 3. Results

Manual dexterity evaluated in the children with UCP using the BBT scale is shown in Table 2, separating the results into the three time points (T1, T2 and T3) measured for the participants, for both the real and virtual versions. The performance on the real scale was significantly greater than that on the virtual version for the dominant upper limb at T1 (*p* = 0.001), T2 (*p* = 0.005) and T3 (*p* = 0.002). However, statistical significance was not reached for the non-dominant upper limb at any time point (*p* = 0.295, *p* = 0.432 and *p* = 0.310, respectively). When all measures of T1, T2 and T3 were mixed, significantly greater performance was detected for the dominant (*p* < 0.001) and non-dominant (*p* = 0.05) upper limbs.

**Figure 3:**
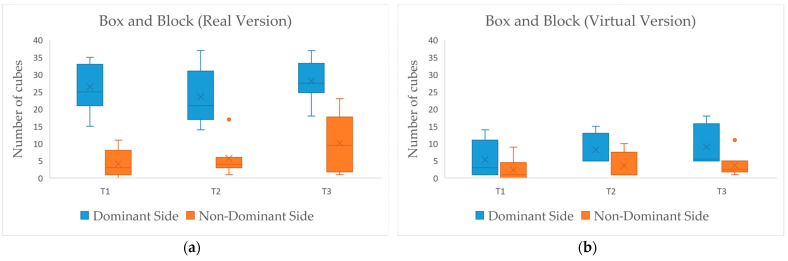
Box plots of BBT scores for the dominant and non-dominant sides. (**a**) Box plots for real BBT scores and (**b**) box plots for virtual BBT scores.

These same results can be observed graphically in the box plots (Figure 3). Participants are able to move a greater number of blocks with the less affected or dominant side, and this difference is more noticeable in the real test than in the virtual version.

### 3.1. Correlation Analysis Between the Real and Virtual BBTs

Spearman’s correlation coefficients between the real BBT and virtual BBT scores are presented in Table 3. There was a moderate correlation between the two tests for the T1 assessment. The correlation coefficients are statistically significant (*p* = 0.028 for the less affected side and *p* = 0.041 for the more affected side) for both sides at time point 2 (T2), with a strong correlation. At the other two time points, there was a moderate correlation in both upper limbs, but the difference was not statistically significant.

The results of the reliability analysis indicated excellent ICCs for the real BBT (0.958; 95% CI: 0.890–0.987) and virtual BBT (0.968; 95% CI = 0.907–0.991) between the different time points of each evaluation.

#### Clinical Analysis

With respect to the clinical variables of strength and hypertonia, we focused on the most affected upper limb and observed that the participants had stable measurements, with no significant differences among the three measurement time points, as shown in Table 4.

For the correlations between the clinical variables and the real and virtual BBT scales, the three measurements of each participant were taken into account. As shown in Table 5, there are correlations between the strengths of the wrist flexors, wrist extensors, finger flexors and finger abductors and the real BBT on the most affected or non-dominant side, whereas the positive correlations between the virtual BBT and only the wrist flexors and finger flexors are statistically significant.

## 4. Discussion

The results of this study suggest that the virtual version of the BBT using the LMC is feasible for application in children with UCP. Previously, the virtual BBT has been validated in a non-pathological population and in people with cervical spinal cord injury to assess upper limb dexterity [22]. However, there is no evidence of studies with this type of virtual application to assess motor dexterity in the pediatric population. The findings here revealed greater performance by participants with UCP in the real BBT than in the virtual BBT, with a moderate correlation between the scores of both tests.

Although a strong correlation could be expected between two tests that assess the same construct, previous studies have also shown moderate correlations between non-immersive [28] or immersive [15,29] virtual BTT and the real BTT. However, other studies have indeed reported strong correlations for non-immersive [22,30] and immersive [21] virtual BTT. With this information, it is difficult to determine whether the key to a stronger correlation with the real BTT is immersion, the pathology of the studied participants or other unknown factors. Although previous studies have validated the virtual BTT with similar correlation results to those in our study, there is a possibility that the failure to find a strong correlation could be due to the fact that the virtual BTT and the real BTT may be assessing different constructs. More studies are needed to definitively validate the virtual BTT according to the real BTT.

In accordance with the results, the literature shows that both people with neurological pathologies and healthy people obtain higher scores on the real scale than on the virtual scale. This difference is usually between 2.5 times and 2.7 times greater on the real scale than on the virtual scale for people with Parkinson’s disease, for the dominant and non-dominant sides, respectively [15]. Another study reported a score that was double the score on the real scale compared with the virtual scale in participants with and without spinal cord injury [22]. This same trend has also been observed in stroke patients [30]. In all these previous studies, a constant difference seems to be maintained between the real and virtual scores, with moderate correlations between them. The same finding was observed in the present study, where children obtained three times the score on the real scale for the dominant side and two times the score for the non-dominant side.

One possible explanation for these findings is the lack of information obtained through non-immersive applications such as those developed from LMC. Non-immersive virtual reality does not provide realistic 3D visual feedback of the participant’s hand position, which could translate into a decrease in dexterity due to the lack of a spatial dimension. Even so, studies that have used immersive VR devices for the BBT continue to obtain scores between 30 and 40% lower in the virtual version than in the real scale [21]. The new trend is to create a hybrid environment that combines real objects with virtual environments, which is known as mixed reality. Currently, only one study has compared the BBT scale in different virtual reality modalities in stroke patients, where it was observed that the BBT test with mixed reality was strongly correlated with the real scale. However, the scores with mixed reality were still 30% lower than with the real BBT scale [31]. Therefore, this area still requires extensive research.

On the basis of the real BBT score, the average age of the population in our study was 5 years, and over the three time points, an average of 25.95 blocks with the dominant hand and 6.50 blocks with the non-dominant hand were performed. If we look at the normality data in the pediatric population without pathology reported by Jongbloed-Pereboom et al., 5-year-old children should be in a range of 27–56 blocks [32]. Therefore, the less affected upper limb is not far from the lower limit of this range; however, in the more affected upper limb, there is a notable decrease in the number of blocks.

When this comparison is carried out in children with UCP who have used the BBT, we can find studies where the most affected side achieved results of 24 blocks [9] and studies where the most affected upper limb obtained an average of 11.8 blocks [33]. Thus, our population continues to score below these results, which could be due to our participants having severely impaired grasping ability, and it has been shown that the results of the BBT greatly depend on the grasping capacity of the non-dominant upper limb [9]. Another aspect that could influence the results is the age of the sample; there is a significant increase in BBT performance that depends on the participant’s age, and this increase is more pronounced between 3 and 8 years, whereas between 8 and 10 years the scores are relatively more stable [32].

An analysis of the average number of blocks passed with the virtual BBT reveals that the scores are much lower, with 7.47 blocks for the dominant side and 3.18 blocks for the non-dominant side. In the case of the dominant side, this difference in scores between the real and virtual BBTs is statistically significant at all three time points. Otherwise, on the non-dominant side, the participants already had very low scores on the real scale, which cannot worsen much on the virtual version. Thus, this virtual scale can have a floor effect for participants with moderately affected gross motor dexterity (MACS levels II and III), where mentioned impairment of manual dexterity would have to do with sensory processing and the MACS level, since the higher the MACS level is, the greater the sensory impairment, and therefore the worse the manual performance [34]. In this case, we do not have references for the pediatric population with a virtual BBT scale, so these differences are probably due to age, as occurs with the real scale.

On the other hand, the difference between the results obtained between the real BBT and the virtual BBT in our study population could also be due to the perceptual–visual impairment that hinders upper limb coordination, reducing performance and impacting the execution of activities of daily living [35]. This impairment directly affects the sequential visual memory of movement, which is crucial for planning movement during the execution of functional tasks [36]. We should also consider other differences in the execution of both tests, such as the coordination required to cross the midline, the grip on the blocks and the force needed to prevent them from falling, which can explain the higher performance with the real BTT and could be a challenge to definitively validate the virtual BTT.

With respect to the reliability of the measures, the ICC shows excellent consistency for both tests at the different time points carried out in this study. These results are consistent with those of all those studies that analyze the ICC of this scale, whether in the real version [9,32] or in the virtual version [15]. This high consistency indicates that, although the measurements were spaced out over a significant period, the virtual BBT can be a reliable method for assessing the gross motor dexterity of the upper limbs in children with UCP. These results are similar to those obtained in the virtual version by Oña et al., although they performed virtual test repetitions on the same day. However, despite spending at least three weeks between measurements, obtaining moderate reproducibility in both measurements suggests that the virtual BBT could also be a reliable test to measure the evolution of upper limb motor dexterity in children with UCP, being a friendly and more attractive method than the real scale [15].

As a secondary objective of the study, a relationship was observed between the strength of the muscles of the most affected or non-dominant side and dexterity, as evidenced by a positive correlation between the strength of the wrist flexors, wrist extensors, fingers flexors and finger abductors and the real BBT. However, only the positive correlation between virtual BBT and wrist flexor strength and finger flexor strength was statistically significant. These correlations may be due to a decrease in hand strength, which can contribute to functional deficits and vice versa [37]. Findings such as muscle power and motor control are strongly related to upper limb function in children with UCP, suggesting that muscle weakness is a key element in the rehabilitation process because of its crucial relationship with manual ability [38]. For all these reasons, it is logical that children with greater strength in the upper limb muscles also obtain better results in gross motor dexterity measured with the BBT, both real and virtual.

The use of the LMC is also a reliable method for the rehabilitation of hand function in children with UCP [31]. It is also worth highlighting that a further potential application of this virtual BBT development could be its use as a training and rehabilitation tool for children with UCP in a gamification context. Previous studies have shown that VR-based interventions are an appropriate method to improve gross and fine motor functions of the upper limbs, as well as to enhance independence in activities of daily living in children with UCP [10,39]. Thus, this represents another potential line of research derived from this pilot study.

This study has a great limitation and that is that the sample of participants with UCP was quite limited, and the ages were highly heterogeneous. Our aim was to determine whether the application of the virtual version of the BBT was viable in a pediatric population with UCP. These initial results show that the scale is applicable to this population, so future studies with a larger group of children with UPC, stratified by age and with a control group, should be carried out to validate the virtual version of the BBT and analyze the feasibility of this tool in assessing and treating this neurological condition. Another possible limitation of the study is the use of non-immersive virtual reality technology. As previously explained, this could affect the test results due to the lack of depth perception. Therefore, in future studies, it would be beneficial to develop the test using immersive virtual reality technology to compare which type of device achieves a higher correlation with the real test and consider the possibility of studying the practice effect with both tests.

## 5. Conclusions

In conclusion, this pilot study revealed moderate correlations between the real scale of the BBT and its virtual version developed with the LMC, demonstrating excellent consistency for a pediatric population of children with UCP. These findings indicate that the virtual BBT can be a valid tool for assessing gross motor dexterity in children with UCP. Additionally, the results revealed a moderate correlation between both scales and the participants’ muscle strength, indicating that greater muscle weakness results in worse gross motor dexterity scores.

## Figures and Tables

**Table 1:** Clinical data of the participants.

Participant	Age (Years)	Sex (M/F)	Diagnosis CP	Affectionate Side (R/L)	GMFCS Level	MACS Level
1	8	M	UCP Spastic	L	II	III
2	6	M	UCP Spastic	R	I	II
3	4	M	UCP Spastic	R	I	II
4	4	M	UCP Spastic	R	I	II
5	4	M	UCP Spastic	R	I	II
6	4	M	UCP Spastic	R	I	II
7	6	M	UCP Spastic	R	I	III
(m ± SD)	5.1 ± 1.6	-	-	-	-	-

F, female; GMFCS, Gross Motor Function Classification System; L, left; M, male; MACS, Manual Ability Classification System; m, mean; R, right; SD, standard deviation; UCP, unilateral cerebral palsy.

**Table 2:** Performance obtained in the BBT (real and virtual modes) for the dominant and non-dominant sides. Data are expressed as the means and standard deviations.

	Box and Block Test (Number of Cubes) Real Test			Box and Block Test (Number of Cubes) Virtual Test		
	T1	T2	T3	T1	T2	T3
Dominant (less affected side)	26.43 ± 7.26	23.57 ± 8.00	28.17 ± 6.31	5.33 ± 5.39	8.20 ± 4.60	9.00 ± 5.90
Mean ± SD	25.95 ± 7.14			7.47 ± 5.29		
Non-Dominant (more affected side)	4.14 ± 3.98	5.71 ± 5.23	10.17 ± 8.38	2.33 ± 3.51	3.60 ± 3.98	3.67 ± 3.67
Mean ± SD	6.50 ± 6.21			3.18 ± 3.52		

**Table 3:** Spearman’s correlation coefficient between real BBT and virtual BBT.

	BBT Real–BBT Virtual		
	T1	T2	T3
Dominant (less affected side)	0.647	0.918 *	0.708
Non-Dominant (more affected side)	0.638	0.894 *	0.530

* Significant difference at *p* < 0.05.

**Table 4:** Clinical measures for the more affected side.

	Non-Dominant (More Affected Side)		
*Strength Measure*	*T1*	*T2*	*T3*
Elbow flexors	4 (4–4)	4 (4–4.75)	4 (4–4)
Elbow extensors	3 (3–4)	3.5 (3–4)	3 (3–3.75)
Wrist flexors	0 (0–2)	1.5 (0.25–2.75)	1.5 (1–2.75)
Wrist extensors	1 (0–1)	1 (0.25–1)	1 (0.25–3.25)
Finger flexors	1 (1–3)	2 (1.25–3.5)	3 (2.25–3.75)
Finger abductors	1 (0–2)	1 (1–2.5)	2 (0.25–3)
*Hypertonia*			
Elbow pronators	1 (0–1)	0 (0–0.75)	1 (0.25–1.75)
Wrist flexors	1 (0–1)	0.5 (0–1)	0 (0–1.5)
Finger flexors	0 (0–0)	0 (0–0)	0 (0–0)

Data are expressed as median and interquartile range.

**Table 5:** Spearman’s correlation coefficients between the real BBT and virtual BBT and the clinical measures of the non-dominant side.

Non-Dominant (More Affected Side)	Box and Block Real Test		Box and Block Virtual Test	
*Strength Measure*	**r**	***p*** **-Value**	**r**	***p*** **-Value**
Wrist Flexors	0.522	**0.022**	0.758	**<0.001**
Wrist Extensors	0.465	**0.045**	0.369	0.145
Finger Flexors	0.798	**<0.001**	0.602	**0.011**
Finger Abductors	0.658	**0.002**	0.217	0.402

*p*-value in bold means that it is statistically significant.

## Data Availability

Data can be obtained by contacting the corresponding author.

## Supplementary Materials

The following supporting information can be downloaded at: https://www.mdpi.com/article/10.3390/jcm14020391/s1, Video S1: Example of performing the real and virtual BBT test.
